# Opportunities for applying whole‐cell bioreporters towards parasite detection

**DOI:** 10.1111/1751-7915.12604

**Published:** 2017-01-26

**Authors:** Alexander J. Webb, Richard Kelwick, Paul S. Freemont

**Affiliations:** ^1^ Centre for Synthetic Biology and InnovationImperial College LondonLondonSW7 2AZUK; ^2^ Section of Structural BiologyDepartment of MedicineImperial College LondonLondonSW7 2AZUK

## Abstract

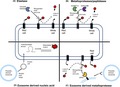

In nature, living cells and organisms have evolved, over billions of years, an astonishing suite of mechanisms that are used to detect and respond to diverse, transient and long‐term external stimuli (Khalil and Collins, 2010). Whole‐cell bioreporters (WCBs; sometimes referred to colloquially as whole‐cell biosensors) are characterized as cells (termed chassis) that have been deliberately modified to make use of re‐engineered versions of these biosensing systems, such that biological detection is coupled with a measurable response. Whilst WCBs have been successfully employed to detect a wide range of environmental pollutants (Tauriainen *et al*., 1997, 1998; Ivask *et al*., 2009; Joshi *et al*., 2009; de Mora *et al*., 2011; Roda *et al*., 2011), metabolites (Sticher *et al*., 1997; Sevilla *et al*., 2015) and other molecules (Hansen and Sørensen, 2000; Urban *et al*., 2007; Chappell *et al*., 2013)(Wu *et al*., 2000), there are clear opportunities for applying WCBs within global health contexts. In parallel, the field of synthetic biology, which uses engineering design principles for constructing novel biological systems and cells, is poised to enable a rapid increase in next‐generation WCBs, which can be applied to global health challenges (Rooke, 2013; Slomovic *et al*., 2015).

Notable examples of microbial WCBs that are currently being integrated into global health initiatives include two arsenic bioreporters, both of which are currently undergoing field trials to determine their utility for detecting arsenic‐contaminated freshwater. One of these is based upon lyophilized *Escherichia coli* (Siegfried *et al*., 2012) cells. The other arsenic bioreporter is based upon engineered *Bacillus subtilis* cells – the researchers are currently seeking regulatory approval as a contained genetically engineered microorganism (European Union directive 2009/41/EC) before moving to widespread field trials of the WCB (see http://www.arsenicbiosensor.org). The decision to seek regulatory approval before use is a direct consequence of applying responsible research and innovation approaches in synthetic biology (Anderson *et al*., 2012), where societal implications and consequences are considered before implementation. Using this process, the *B. subtilis* project identified complex social, cultural and data protection issues that interrelated with the technological development and implementation of the arsenic WCB. One example was the need to respond to the concerns of Nepalese villagers, during initial field trials, by changing the colour output of the bioreporter test, so that green signified that the water was OK, and shades of indigo were set to a numerical scale for progressively higher levels of arsenic contamination. However, beyond such concerns, critics of WCBs often raise concerns relating to the risks associated with accidental or even deliberate release of engineered WCB organisms. The decision to use *B. subtilis* as a WCB in the arsenic biosensor project was partly influenced by the United States Food and Drug Administration (U.S. FDA) classification of *B. subtilis* as a generally regarded as safe (GRAS) organism (Cutting, 2011). Whilst GRAS is a term that specifically relates to food applications, it is arguable whether the use of GRAS organisms has any bearing on whether engineered forms of GRAS organisms (e.g. WCBs) pose fewer safety risks than non‐GRAS organisms. Risks associated with WCBs may also be mitigated through the continual development of novel physical containment (e.g. sealing the WCBs within a capsule) and genetic containment (e.g. genetic kill switches, synthetic auxotrophy (Wright *et al*., 2015; Mandell *et al*., 2015)) strategies – such approaches make the safe use of WCBs more practical. Therefore, in combination, these safety strategies and societal considerations will in the near future make it much more feasible to utilize WCBs more widely. Essentially, we argue that it is currently difficult to utilize WCBs in society because their successful implementation is dependent upon the interplay between their technological development and the practicalities of their usage. These implementation practicalities must also include an understanding of the social, political and economic factors of the context in which the WCB will be used. This type of responsible research and innovation is integrated into the new field of synthetic biology (Kelwick *et al*., 2014) where the design of the engineered biological system (e.g. WCB) includes activities to connect and engage with the downstream stakeholders of the technology being constructed.

The field of synthetic biology is also enabling researchers to build novel and more complex types of engineered biological systems, many of which can be implemented as WCBs. Several excellent reviews have been written that describe the strategies used to construct and test different types of bioreporter mechanisms (e.g. transcriptional, translational and post‐translational circuits) in engineered WCBs (Khalil and Collins, 2010; Goers *et al*., 2013; Kopniczky *et al*., 2015). There are also novel types of WCBs that do not rely on a genetic circuit to provide the sensing per se. Instead, these novel bioreporters utilize a protein or fusion protein that is exposed to the external environment outside of the cell. When these proteins interact with the metabolite, molecule or protein biomarker that they are designed to detect, modifications such as activation or cleavage can be enabled. As such, external exposure of the detector protein on the surface of these types of WCBs confers the ability to detect exogenous analytes or protein biomarkers. As some of the biomarkers can be derived from other living organisms (e.g. proteases), we believe that correctly designed WCBs are highly suited for the detection of parasites. Whilst there are many types of parasitic biomarkers, we argue that parasite‐derived proteases are a particularly relevant class of biomarkers for detection. Proteases are a biological signature for many parasitic diseases, and proteases are important in many parasitic physiological processes (McKerrow *et al*., 2006). For instance, proteases are secreted by parasites and their eggs to invade the surrounding tissues and digest the local environment to provide food. Thus, there are numerous potential targets for the detection of different parasites – which could be exploited using WCBs as cheap, rapid and effective diagnostic or epidemiological tests for diseases and global health settings where there is the greatest need, e.g. neglected tropical diseases (NTDs). However, the ubiquitous presence of parasitic proteases also presents a challenge for protease‐based bioreporters in that parasitic samples contain a complex cocktail of proteases, some of which may elicit off‐target effects through less‐discriminate cleavage. To mitigate these risks, synthetic biology approaches, such as DNA sequence optimizations, could be used to change amino acids as a means to eliminate, where possible, off‐target protease recognition motifs from fusion protein designs. Moreover, if modularity is introduced into these designs, then it is possible to easily change the recognition motif to incorporate a sequence that has been validated as conferring a high level of specificity to the target parasite protease.

As an exemplar, schistosomiasis (bilharzia) is a debilitating disease caused by the waterborne schistosoma parasite. Estimates have suggested that over 200 million people worldwide are affected by schistosomiasis (WHO, 2002; Gryseels *et al*., 2006) and annual mortality rates are thought to be upwards of 280, 000 people in sub‐Saharan Africa alone (Gryseels *et al*., 2006). There is thus an urgent need for a rapid, cheap and specific test that can detect for schistosoma either *in situ* at water sites or in makeshift laboratories near water courses. The current gold standard diagnostic technique for schistosomiasis is the microscopic examination of patient excreta for the detection and identification of parasitic eggs (Gryseels *et al*., 2006). The WHO recommends the Kato‐Katz faecal examination technique (Katz *et al*., 1972), the use and microscopic examination of polycarbonate filters for eggs in the urine, or the urine haem dipstick assay (WHO, 1993; Colley *et al*., 2014). Alternative methods, such as oil‐laced traps (Ahmed *et al*., 2002) or mouse bioassays (Spear *et al*., 2004), can be used *in situ* to survey for the presence of infective schistosomes. However, there can be a considerable time lag between infection and observation of possible infection using some of these techniques. Furthermore, antibody‐based detection techniques may have cross‐reactivity to other helminth parasites, which limits their usefulness for parasite species identification (Ross *et al*., 2002; Doenhoff *et al*., 2004; Stothard *et al*., 2009). Molecular analyses such as polymerase chain reaction (PCR; Lodh *et al*., 2014; Hung and Remais, 2008), 16S ribosomal RNA (rRNA; Mach *et al*., 2015) and loop‐mediated isothermal amplification (LAMP; Fernández‐Soto *et al*., 2014), whilst more specific, all require, to a degree, trained people, laboratory equipment, access to refrigeration and reagents, which limits their accessibility in resource‐limited settings. In contrast, WCBs are self‐replicating and thus are self‐renewing giving a sustainable supply of the bioreporter test. Additionally, WCBs using bacteria can be lyophilized enabling their transport and application at ambient temperatures. Most usefully, at the point‐of‐use they can be designed to give simple outputs to aid ease‐of‐use for non‐trained individuals by providing, if appropriate to its implementation, a simple yes/no test based on a colorimetric output.

To demonstrate these advantages, we recently designed and tested several WCBs that were engineered to detect *Schistosoma mansoni*, one of the causative agents of schistosomiasis (Webb *et al*., 2016). In our study, we used both *E. coli* and *B. subtilis* as the WCB chassis, which were designed to detect the presence of the elastase enzyme released by the cercarial larvae, as part of the infection process of primary hosts – such as humans (Gryseels *et al*., 2006). The elastase is released and facilitates invasion by degrading the dermal elastin (Salter *et al*., 2000). Our WCBs possess a modular design, whereby an anchor protein holds the biosensor component on the external surface of the cell. This anchor is fused to a flexible linker module, which also comprises the cercarial elastase peptide recognition motif, which is then further fused to a tag module that can be antibody‐labelled (Fig. 1A; Webb *et al*., 2016). Thus, in the presence of viable cercariae the elastase recognition motif of these WCBs will be cleaved and, via the release of the antibody‐labelled tag results in a detectable loss of colour, in the presence of the parasite (Fig. 1A). Our laboratory results were promising though after discussions with stakeholders we have gained a deeper appreciation of the challenges of implementing our WCBs in real‐world settings and conditions. One important issue that must be considered is the schistosoma cercarial detection limit of the WCBs. We previously considered what the detection limit might be in terms of the amount of elastase released by each individual cercarial larva and based upon our analyses we estimated that each larva produces elastase in the pg range (Webb *et al*., 2016). Therefore, we concluded that as the level of cercariae present in infected water courses can vary greatly, we may need to concentrate the number of cercariae present in the volume of water sample tested to enable detection using our WCBs. A proven strategy to overcome this challenge is to use a schistosoma trap system (Shiff *et al*., 1993). The trap design incorporates a glass slide, which is coated with stimulant matrix comprising linoleic acid in clear nail varnish (Shiff *et al*., 1993). Linoleic acid is a known chemoattractant for schistosoma cercariae and, along with physical contact against the trap surface, induces release of the head gland contents including elastase (McKerrow and Salter, 2002). New designs of this trap have replaced the linoleic acid component with natural oils high in polyunsaturated fatty acid content, such as sesame oil, which can be sourced locally to the regions to be tested (Ahmed *et al*., 2002). This flexibility in trap design may enable acceptance when applied locally in different geographical regions within the context of responsible research and innovation. For instance, local supplies of the fatty acid may provide economic opportunities to the affected communities. There are also other alternative trap approaches to capture different parasites, which could potentially be adapted to feed into and implemented in WCB strategies (Paul *et al*., 1981; Velo *et al*., 2016).

**Figure 1:**
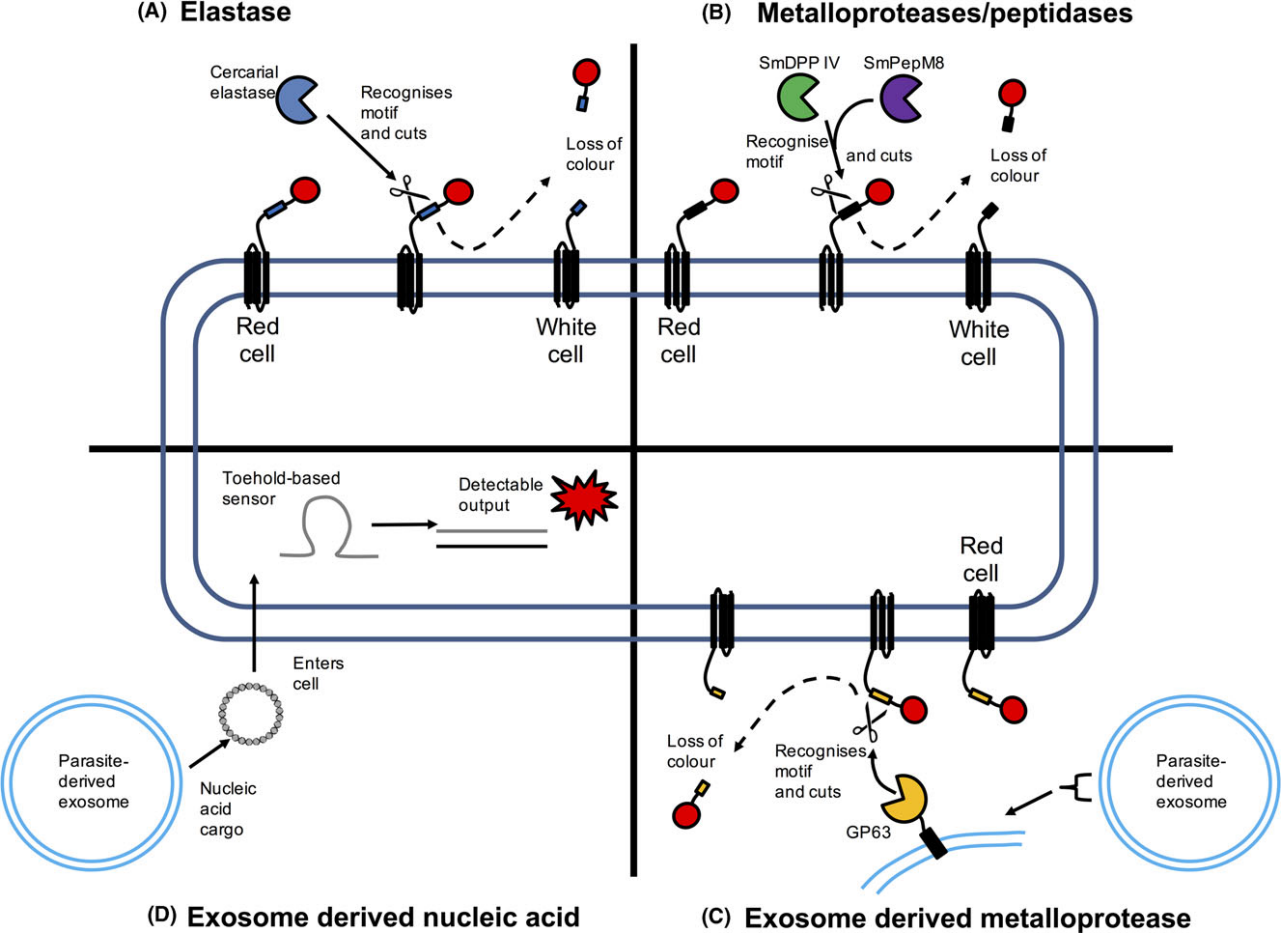
Possible strategies for whole‐cell bioreporter detection of parasitic biomarkers. Detection of parasite‐derived proteases such as (A) elastase or (B) metalloproteases/peptidases secreted from schistosoma cercariae during invasion. In these examples, in the presence of the parasite‐derived protease, the tag/detectable output is cleaved at a specific recognition motif within the biosensor component of the WCB. This results in a detectable loss of colour. Parasites including *Leishmania* produce exosomes. Proteases such as (C) GP63 are anchored in the membranes of *Leishmania*‐derived exosomes and could be detected using similar WCB designs to that described in (A) and (B). Alternatively, parasite‐specific nucleic acids, including those which are found within parasite‐derived exosomes, could be detected using WCBs that incorporate genetic circuits such as (D) Toehold RNA aptamers that change confirmation in the presence of a defined RNA sequence and enable translation of a reporter protein.

Also in the context of responsible research and innovation, we may have to be flexible in terms of the colour output of the schistosoma WCBs. The modular nature of our WCBs design means that we can readily replace the current output with alternatives that are more easily observable with the naked eye, or as noted by the arsenic biosensor project use outputs that take into account cultural sensitivities around colour. We can use a multitude of DNA assembly techniques, e.g. Golden Gate Assembly, to achieve this (Kelwick *et al*., 2014). These techniques can also be used to change the recognition motif of our reported WCB design to any other protease target. For instance, it is conceivable to detect other proteases released by the cercariae by designing different protease recognition motifs, which can be easily incorporated into the WCB design. Other examples of schistosoma‐related proteases that WCBs could be designed to target include the metalloprotease SmPepM8 and the dipeptidyl peptidase IV (SmDPP IV), both of which have been detected in the secretory glands of cercariae (Fig. 1B; Curwen *et al*., 2006). Indeed, these two enzymes have been suggested as having a role in invasion of the primary host (Curwen *et al*., 2006). Novel WCBs could also be designed to detect other parasitic NTDs including leishmaniasis, which is caused by infection with the parasitic protozoan *Leishmania*, spread by the bite of phlebotomine sand flies. The *Leishmania* reside in the sand fly midgut and secrete exosomes, which are co‐egested with the parasite during the sand flies blood meal (Atayde *et al*., 2015). Exosomes are small lipid vesicles formed within multivesicular bodies and are in the size range 50–100 nm in diameter (Silverman and Reiner, 2011). Interestingly, a number of different markers and proteins are present on the surface of parasite‐derived exosomes, including the metalloprotease GP63 (Silverman and Reiner, 2011; Atayde *et al*., 2015), which is known to cleave host intracellular proteins (Hallé *et al*., 2009). It could thus be possible to design WCBs that target GP63 or other proteases present on the surface of the *Leishmania* exosomes (Fig. 1C). Alternatively, WCB designs could be used to detect the unique nucleic acid cargos that are present within exosomes (Fig. 1D). Nucleic acid exosome biomarkers have already proven to be useful in the field of cancer biology (Wendler *et al*., 2016) and it is conceivable that synthetic genetic circuits that incorporate RNA aptamers (e.g. toehold RNA switches) could be designed and implemented within WCBs for the purposes of detecting parasite‐derived exosome nucleic acid signatures (Fig. 1D; Pardee *et al*., 2014; Kopniczky *et al*., 2015). However, the ubiquitous nature of parasite‐derived proteases, their abundance and role in host invasion does make them a more logical target for WCBs. Regardless of the specific parasite target, we believe that based upon existing and newly discovered parasite biomarkers (e.g. parasite‐derived proteases) coupled with novel WCB designs (e.g. surface‐exposed fusion proteins with specific recognition motifs), that a broad array of novel and inexpensive WCBs could be rapidly developed to detect a wide range of parasites. We therefore envision in the near future that WCBs will play a pivotal role in detecting parasites and other animal/human pathogens and that such bioreporters will be used across several contexts including in the field, in clinics and in the home. We would argue that the implementation of a new generation of cheap and inexpensive bioreporters enabled by responsible research and innovation approaches in synthetic biology will make a transformative impact in tackling global health.

## Conflict of interest

The authors declare no competing financial interests.
